# Modernising the Mental State Examination: embedding eating and nutritional assessment into the 21st-century MSE

**DOI:** 10.1192/bjb.2026.10213

**Published:** 2026-08

**Authors:** Edwin Birch, James Downs, Agnes Ayton

**Affiliations:** 1 Psychiatry, Oxford Health NHS Foundation Trust, Oxford, UK; 2 Independent scholar; 3 Cotswold House, Oxford Health NHS Foundation Trust, Oxford, UK

**Keywords:** Mental State Examination (MSE), feeding or eating disorders (FEDs), nutritional psychiatry, psychiatric assessment, medical education

## Abstract

**Aims and method:**

The Mental State Examination (MSE) is a core component of psychiatric assessment and medical training, yet it was developed before feeding or eating disorders (FEDs) were widely recognised. FEDs are now common, clinically severe and frequently missed in routine assessments. We conducted a narrative review of the historical development of the MSE, current UK medical education standards and relevant literature, supplemented by a lived-experience perspective, to examine whether the MSE adequately captures eating behaviour, nutritional status and body image disturbance.

**Results:**

We identified no published studies examining the explicit inclusion of FED-related psychopathology within the MSE. Current frameworks lack systematic prompts for eating behaviours and nutrition, contributing to under-recognition. Contributing factors include historical MSE design, limited curriculum coverage, clinician uncertainty and patient non-disclosure.

**Clinical implications:**

Embedding brief, semi-structured prompts into the MSE is a feasible training-aligned approach to improve detection, support curriculum modernisation and enhance patient safety.

Since its widespread adoption in the second half of the 20th century, the Mental State/Status Examination (MSE) has been the primary method for systematic assessment of psychopathology across general psychiatry, primary care and hospital medicine.^1^ It is widely used as part of assessment, alongside a full psychiatric and medical history, physical examination and laboratory investigations, to formulate a differential diagnosis.

The MSE encompasses both observable signs (including psychomotor retardation, disordered speech and abnormal affect) and reported symptoms (including auditory hallucinations, guilt or suicidal ideation) of mental disorder, thereby providing a structured assessment of the individual’s mental state at a given point in time. The MSE’s central role in psychiatric practice has secured its place in both undergraduate and postgraduate medical training, as well as in clinical examinations worldwide.^2,3^ However, it was developed in an era when eating disorders were rare, with its traditional focus remaining on mood, psychosis and cognition.

The recently updated ICD-11 recognises three primary eating disorders: anorexia nervosa, bulimia nervosa and binge eating disorder.^4^ This is set alongside a family of so-called feeding disorders, which include avoidant-restrictive food intake disorder, rumination–regurgitation disorder and pica.^4^ An additional diagnostic category exists for other specified feeding or eating disorders. These distinct diagnoses are collectively referred to as ‘feeding or eating disorders’ (FEDs) in ICD-11, a diagnostic system largely (but not completely) mirrored in the American DSM-5.^5^

The frequency of FEDs has risen markedly over recent decades, with further escalation observed both during and following the COVID-19 pandemic.^6,7^ Although high-quality incidence studies remain limited, available population- and register-based data indicate increases in newly diagnosed FEDs, particularly among children and adolescents.^6,8^ In clinical practice, however, prevalence is the more relevant measure because it reflects the number of people currently requiring recognition and care. Recent UK and international data show substantial and growing prevalence across major FED categories, contributing to increased clinical demand.^6,8^ FEDs now represent a significant and expanding source of psychiatric morbidity, and are consistently associated with elevated morbidity, mortality and global disease burden.^9^ This reinforces the need for systematic inquiry within routine psychiatric assessment.

Despite their severity and complexity, FEDs remain under-recognised in routine clinical practice, especially when assessments rely on the traditional MSE, which does not prompt clinicians to explore weight and shape concerns, disordered eating behaviours or nutritional status.^1^ This can result in delayed or missed diagnoses, with serious consequences for patient safety. This gap in detection is further compounded by the limited attention given to FEDs in medical education and training,^10^ despite repeated calls for reform.

In 2017, the UK Parliamentary and Health Service Ombudsman report, ‘Ignoring the alarms: How NHS eating disorder services are failing patients’, identified systemic failures in the recognition and treatment of eating disorders, attributing preventable deaths in part to inadequate medical training.^11^ The Public Administration and Constitutional Affairs Committee echoed these concerns, calling for urgent improvements in medical education to safeguard patients.^12^ Since then, progress has been minimal and subsequent coroners’ reports and academic publications have continued to highlight similar failings, underscoring the persistent risk posed by insufficient training and outdated assessment tools.^13,14^

In light of this, it remains more important than ever for medical educators to question whether nutritional psychopathology is appropriately captured in the current MSE framework; ensuring that FEDs are not neglected in routine psychiatric assessment.

## Aims

In this paper we examine whether the MSE is adequate in its current format, in particular regarding its emphasis on the assessment of nutritional status and FEDs. We consider how the MSE could be updated to better reflect changing patterns of mental disorders and meet contemporary clinical needs.

## Method

We conducted a narrative overview of the historical development of the MSE and current UK medical education documents (Royal College of Psychiatrists (RCPsych) curricula and General Medical Council (GMC) standards). We undertook a PubMed search of the literature to identify relevant articles and, due to the wide historical breadth in the origins of the MSE, relevant resources were also derived from articles in the initial literature search alongside references from primary texts (please refer to Appendix 1 in the supplementary materials).

A lived-experience perspective is provided by author J.D.

## Results

We identified no published studies examining the explicit inclusion of FEDs within the MSE. Despite its central role in psychiatric assessment and training, the MSE has not evolved to reflect the breadth and diversity of contemporary FED presentations. Several interconnected historical, educational and structural factors appear to contribute to this gap.

### The historical origins of the MSE may explain a lack of clinician confidence in, and emphasis on, FEDs and nutrition

The origins of the MSE are challenging to delineate. The tool was born out of the need for objective psychiatric assessment across diverse clinical environments, which means that the story of the MSE is closely tied to the history of descriptive psychopathology. Karl Jaspers’ *General Psychopathology* (1912) is widely regarded as an early foundation of modern descriptive psychopathology.^15^ However, the structure of the contemporary MSE is more directly influenced by Wing et al’s development of the Present State Examination in the 1970s, which provided a systematic approach to documenting psychiatric signs and symptoms.^16^

At no stage has there been international consensus on the precise content or order of the MSE. Versions in use globally can contain anything from 6 to 18 domains, reflecting the organic process of adaptation opposed to formal development or any Delphi-style methodology.^3^ Drawn from the British Medical Association, the *de facto* UK standard is summarised in Table 1 and is a structure widely replicated in English-language psychiatry textbooks.^1^ Despite its central role, neither the GMC nor RCPsych curricula specify subheadings requiring that clinicians systematically assess eating behaviour, nutrition or weight and shape concerns.^2,17^

**Table 1:** Illustrative examples of how feeding or eating disorder psychopathology can be documented within standard Mental State Examination (MSE) domains Table 1 long description.

MSE domain	Example prompt	Possible clinical observations
Appearance	Observe nutritional status and physical signs	Weight across the full spectrum in both sexes; loose or concealing clothing; Russell’s sign; parotid swelling; visible malnutrition or signs of vitamin deficiencies
Behaviour	Observe response when food or body image is discussed	Avoidance of eye contact; body checking; restlessness or agitation; withdrawal
Speech	Speech could be hesitant when discussing eating- or weight-related topics	Speech abnormalities most often present due to comorbid psychiatric illness such as depression or anxiety
Mood and affect	‘How do you feel about yourself, especially around food or your shape or weight?’	Shame, guilt, anxiety, irritability; low mood; restricted or incongruent affect in relation to physical state
Thought form	‘Do thoughts about food or weight come into your mind when you try to focus on other things?’	Perseveration, rigidity, obsessive ruminations, difficulty shifting attention
Thought content	‘Are there rules you feel you must follow about eating, exercising or your weight?’	Overvalued ideas about weight/shape; intrusive urges to binge or purge; overvalued ideas about weight and shape; avoidance of foods (ARFID)
Perception	‘Do you sometimes feel your body looks different from what others tell you?’	Body image distortion; misperception of body size; heightened discomfort with bodily sensations (e.g. fullness)
Cognition	Observe attentional difficulties, slowed thinking or poor memory	Poor concentration; slowed thinking; memory lapses; indecisiveness, especially if malnourished or electrolyte-imbalanced
Insight	‘How do you view your current eating habits and their impact on your health?’	Anorexia nervosa: denial or minimisation of severity; bulimia nervosa/binge eating disorder: acknowledgement of distress but underestimation of risks; ARFID: focus on sensory sensitivities, little recognition of psychiatric impact

The MSE was developed in 20th-century psychiatric practice at a time when presentations were dominated by psychotic, affective and neurocognitive disorders. This historical context explains the enduring emphasis on hallucinations, delusions, mood and cognition, while systematic inquiry into nutrition and body image was never integrated.^18,19^

Understanding this background is important; it helps to explain the current omission of FEDs from routine assessment and training, and underpins the rationale for updating the MSE to reflect contemporary clinical needs.

These omissions are particularly problematic given the breadth of FED diagnoses, their prevalence among all genders and cultures and their high rates of psychiatric and medical comorbidity.^20^

Research suggests that many clinicians lack confidence and training in assessing eating behaviours and nutritional status.^18,21^ As a result, compounded by gaps in experience and awareness, clinicians might not ask targeted questions about dietary intake, compensatory behaviours or preoccupation with weight and shape or body image concerns, even though these features are clinically relevant across many psychiatric conditions.^18^ This contributes to underdiagnosis at the population level and increases the likelihood that routine MSEs do not include structured inquiry into weight, diet or nutrition.

In addition, the predominantly unstructured delivery of the MSE leads to significant variation in depth and focus, depending on the clinician’s experience and assumptions. This makes the assessment of FEDs particularly vulnerable to omission, especially now that a diagnosis of anorexia nervosa can be made in non-underweight individuals, meaning that previous ‘catch-alls’ of appearance or assessment of weight during physical examination may no longer be adequate.^4^ This risk is especially pronounced for people living with obesity, who have high rates of comorbid eating disorders, and for those with FED, who commonly experience comorbid anxiety, depression, autism or obsessive–compulsive disorder.^20^ These overlapping presentations can shift clinical attention away from FEDs and contribute to missed or delayed diagnosis.

### Patient-related factors often preclude voluntary disclosure

FEDs often remain undetected because the individual may not spontaneously disclose their symptoms. This is particularly true in conditions such as anorexia nervosa, where behaviours are frequently ego-syntonic – that is, aligned with the person’s goals, values or sense of identity. In some cases this can amount to impaired awareness of illness, and individuals will not perceive their behaviours or physical state as problematic.

Even when some awareness is present, the physical consequences of the illness may be interpreted as signs of success or control. Shame, embarrassment and internalised stigma further deter disclosure, particularly when the individual is not directly asked about their experiences, or is from a marginalised group subject to unique stigmas, such as men; people of colour; lesbian, gay, bisexual, transgender and queer/questioning individuals; or those with higher-weight bodies.

As author J.D. reflects, in the context of his own lived experience:

‘One of the most dangerous aspects of my eating disorder was that I didn’t recognise I was unwell. My behaviours felt entirely logical and were directly tied to my goals around weight and shape […] In fact, there were even times when I saw the severe impacts of my illness as some kind of marker of “success”, even if they were putting my life at risk.’

Moreover, malnutrition itself can impair cognition and emotional regulation. It may present as low mood, anxiety or emotional blunting – symptoms that can easily be misattributed to primary mood or anxiety disorders. This complexity is reflected in the high rates of psychiatric comorbidity among people with FEDs (Schmidt et al^20^).

Author J.D. also describes how such complexities shaped his clinical encounters:

‘I was rarely asked whether I avoided social situations involving food, how much time I spent thinking about eating and weight, or whether I felt panic at the idea of breaking my rigid eating rules […] Rather than recognising eating disorder psychopathology, clinicians searched for alternative explanations that aligned with gendered expectations.’

### Structural and systemic factors hinder MSE versatility

These challenges are compounded by structural issues in medical education. Although the MSE is a core component of assessment across medical specialties, there is little formal instruction on how to adapt it for detection of FEDs. Curricula across undergraduate, postgraduate and continuing professional education often marginalise nutrition and disordered eating, despite their relevance across health conditions and psychotropic prescribing.^18^ Without modernising educational content and clinical tools, these gaps in recognition and response are likely to persist. Addressing these shortcomings will require targeted changes to how the MSE is taught, applied and supported in practice.

### Modernising the MSE using a semi-structured approach to nutrition and body image

The results of this review suggest that the pre-existing MSE framework does not require reinvention, but would benefit from thoughtful adaptation to more systematic inclusion of eating behaviours and body image concerns. Table 1 outlines possible examples of semi-structured interview questions, with responses that can be recorded across existing MSE domains – making documentation feasible across clinical settings. These examples are intended as an illustrative scaffold, and they do not replace the detailed exploration of eating behaviours undertaken in the psychiatric history. Furthermore, clinician judgement will still be highly relevant in determining the phraseology of these suggested prompts, timing of inquiry and depth of inquiry to suit the presentation of the individual.

A revised MSE would support, rather than disrupt, the overarching goals of psychiatric assessment. This represents an evolution consistent with the way in which the MSE has historically adapted to encompass emerging areas such as suicidality, cognition and risk. Introducing questions into the MSE regarding nutrition can, in turn, destigmatise and complement details taken separately in the psychiatric history, such as engagement in purging, restriction or other maladaptive practices. Taken together, it can foster a culture where nutrition is recognised as vital for mental health, benefiting all and not only those with FED.

It is important to reiterate that further developments to the MSE should be co-produced with a diverse range of people with lived experience (PWLE) to ensure that adaptations are relevant, acceptable and sensitive to the needs of different populations.

## Discussion

Although the MSE has developed over the past century through tradition, clinical practice and professional consensus, there is now a requirement to update the framework for systematic assessment of nutritional status, eating behaviours and body image.

The GMC, the Parliamentary and Health Service Ombudsman and the Academy of Medical Royal Colleges have all explicitly recognised the urgent need to improve medical education and clinical competence in regard to eating disorders. Repeated inquiries have shown that gaps in recognition contribute directly to delayed diagnosis, preventable deterioration and avoidable deaths. The exclusion of FED-related inquiry from the MSE perpetuates these risks and reinforces stigma and diagnostic bias, amounting to a structural inequity within psychiatric practice.

A proactive, semi-structured approach should be embedded into routine psychiatric assessments in all settings to bring the MSE in line with contemporary clinical needs. Nutritional status, eating behaviours and weight changes are essential for the identification of FEDs and are clinically relevant across a broad range of psychiatric and physical conditions, including those treated with psychotropic medications. Normalising the discussion of diet and nutritional status in psychiatric practice would promote a more holistic and person-centred approach to care. There would also be scope for future refinement of these proposed additions; making use of Delphi or other quality-improvement methods, co-produced with PWLE, would be a logical next step.

Our proposal is a simple, pragmatic and time-feasible enhancement to the MSE. It does not extend the interview length but embeds a brief hierarchical framework of prompts (analogous to those used for mood, psychosis or suicidality) that can be applied flexibly depending on the presentation. This change aligns with the objectives of medical regulators, reduces diagnostic disparity and strengthens patient safety. This small investment of time (requiring no more than 1–2 min where no FED features are present) may prevent delayed diagnosis and crisis presentations, ultimately saving time overall. Incorporation explicitly into the MSE may additionally confer the much-needed benefit of increased attention to FEDs among medical trainers and trainees, beginning to correct the current paucity of training given to the disorders.^10^

Any continued reliance on patients to volunteer their experiences risks missing key clinical information, including potentially life-threatening symptoms. Early identification and intervention are known to improve outcomes and prevent the escalation of physical and psychological morbidity in FEDs.^22^

We also add that inquiry into nutritional psychopathology should not be conflated with administration of formal screening tools such as SCOFF; such tools are best reserved for situations where a disorder is clinically suspected. FED-related prompts within the MSE instead provide a baseline level of inquiry across all psychiatric assessments, and in medical education, without imposing the burden of universal screening questionnaires.

Further concerns that questions about diet and weight may cause offence are not supported by available evidence: PWLE of FEDs report that failing to ask reinforces stigma and delays care whereas sensitive, structured inquiry is often welcomed, much like standardised questioning about suicide risk.^23^ Co-producing MSE revisions with PWLE and caregivers may improve acceptability, reduce stigma and enhance clinical relevance. Although this article’s focus is on medical education at the undergraduate and postgraduate level, we anticipate a secondary benefit of patient-led changes to the MSE for GPs and emergency medicine clinicians – enabling the use of delicate, high-value questions within limited time constraints to support recognition and referral.

Modernising the MSE to routinely prompt inquiry into FED-related psychopathology is therefore a scalable, evidence-informed step toward, improving early detection, patient safety and treatment outcomes. Crucially, it also provides a powerful educational scaffold, equipping future clinicians with the competencies needed to respond to the growing burden and complexity of FEDs.

### Key messages for clinical educators

- Feeding, eating and nutritional psychopathology are relevant across all psychiatric diagnoses, yet remain under-recognised in routine mental state assessments due to structural omissions in current training and practice.
- The MSE lacks explicit prompts to explore eating behaviours, body image disturbance and nutritional status, contributing to missed or delayed detection of FEDs.
- Incorporation of brief, semi-structured questions into the MSE offers a feasible and clinically grounded strategy to improve recognition of FEDs, support diagnostic accuracy and reinforce parity between physical and mental health.
- Updating MSE training within undergraduate and postgraduate curricula is essential to align medical education with national competencies (GMC, RCPsych), public health needs and emerging fields such as nutritional psychiatry.
- Embedding structured nutrition-related inquiry into medical education will reduce stigma, improve clinician confidence and normalise nutritional assessment in both mental and general healthcare.
- Objective Structured Clinical Examination and Clinical Assessment of Skills and Competencies assessments should include eating disorder scenarios and reward systematic assessment of weight, diet and compensatory behaviours – ensuring that these areas become core components of clinical skill development.

Co-production of MSE updates with PWLE will ensure that educational tools are acceptable, inclusive and sensitive to the needs of diverse populations.

## Supporting information

Birch et al. supplementary material Birch et al. supplementary material

## Data Availability

Data availability is not applicable to this article because no new data were created or analysed in this study.

## About the authors

**Edwin Birch** BM BCh, BA, Oxford Health NHS Foundation Trust, Oxford, UK. **James Downs** MEd (Cantab), MSc, expert by experience and independent researcher. **Agnes Ayton** MD, FRCPsych, MMedSc. MSc, Oxford Health NHS Foundation Trust, Oxford, UK.

## Supplementary material

The supplementary material is available online at https://doi.org/10.1192/bjb.2026.10213

## Table 1 Long description

The table has three columns: MSE domain, Example prompt, and Possible clinical observations. It contains 10 rows, each representing a different domain of the Mental State Examination. Row 1: Appearance, Observe nutritional status and physical signs, Weight across the full spectrum in both sexes; loose or concealing clothing; Russell's sign; parotid swelling; visible malnutrition or signs of vitamin deficiencies. Row 2: Behaviour, Observe response when food or body image is discussed, Avoidance of eye contact; body checking; restlessness or agitation; withdrawal. Row 3: Speech, Speech could be hesitant when discussing eating- or weight-related topics, Speech abnormalities most often present due to comorbid psychiatric illness such as depression or anxiety. Row 4: Mood and affect, How do you feel about yourself, especially around food or your shape or weight?, Shame, guilt, anxiety, irritability; low mood; restricted or incongruent affect in relation to physical state. Row 5: Thought form, Do thoughts about food or weight come into your mind when you try to focus on other things?, Perseveration, rigidity, obsessive ruminations, difficulty shifting attention. Row 6: Thought content, Are there rules you feel you must follow about eating, exercising or your weight?, Overvalued ideas about weight/shape; intrusive urges to binge or purge; overvalued ideas about weight and shape; avoidance of foods (ARFID). Row 7: Perception, Do you sometimes feel your body looks different from what others tell you?, Body image distortion; misperception of body size; heightened discomfort with bodily sensations (e.g. fullness). Row 8: Cognition, Observe attentional difficulties, slowed thinking or poor memory, Poor concentration; slowed thinking; memory lapses; indecisiveness, especially if malnourished or electrolyte-imbalanced. Row 9: Insight, How do you view your current eating habits and their impact on your health?, Anorexia nervosa: denial or minimisation of severity; bulimia nervosa/binge eating disorder: acknowledgement of distress but underestimation of risks; ARFID: focus on sensory sensitivities, little recognition of psychiatric impact.

Navigate back to Table 1.
