# Supplementary material for: Modernising the Mental State Examination: embedding eating and nutritional assessment into the 21st-century MSE
Source: BJPsych Bull. 2026 Feb 23;50(4):355–9. doi: 10.1192/bjb.2026.10213 (PMC13386287; doi:10.1192/bjb.2026.10213)
Supplement: Birch et al. supplementary material [file S2056469426102137sup001.docx]

# Appendix 1

Literature search methodology

1. **The historical basis and emphasis of the MSE**

Primary texts utilised:

Oyebode F. *Sims’ symptoms in the mind: textbook of descriptive psychopathology*. 6th ed. Edinburgh: Elsevier 2022

*Pubmed* search terms:

(((Mental state examination[Title/Abstract]) OR (mental status examination[Title/Abstract])) NOT (mini mental state examination[Title/Abstract])) AND (History)

191 abstracts generated for the years between 2005-2025 inclusive. Abstracts were screened with 7 being relevant to subject material

1. **Clinician confidence in themes of nutrition in psychiatric interview**

*Pubmed* search terms:

((psychiatry[Title/Abstract] OR "mental health"[Title/Abstract]) AND (nutrition[Title/Abstract] OR diet[Title/Abstract] OR "nutritional assessment"[Title/Abstract] OR "dietary advice"[Title/Abstract])) AND (confidence[Title/Abstract] OR attitudes[Title/Abstract] OR knowledge[Title/Abstract] OR "self-efficacy"[Title/Abstract] OR "clinical practice"[Title/Abstract]) AND (“psychiatrist” [Title/Abstract] OR "mental health nurse"[Title/Abstract] OR "healthcare professional"[Title/Abstract])

23 abstracts generated for the years 2005-2025 inclusive. Abstracts were screened with 4 being relevant to subject material.
